# Diagnostic value of a plasma microRNA signature in gastric cancer: a microRNA expression analysis

**DOI:** 10.1038/srep11251

**Published:** 2015-06-10

**Authors:** Xin Zhou, Wei Zhu, Hai Li, Wei Wen, Wenfang Cheng, Fang Wang, Yinxia Wu, Lianwen Qi, Yong Fan, Yan Chen, Yin Ding, Jing Xu, Jiaqi Qian, Zebo Huang, Tongshan Wang, Danxia Zhu, Yongqian Shu, Ping Liu

**Affiliations:** 1 Department of Oncology, First Affiliated Hospital of Nanjing Medical University, 300 Guangzhou Road, Nanjing 210029, PR China; 2 Department of Pathology, First Affiliated Hospital of Nanjing Medical University, 300 Guangzhou Road, Nanjing 210029, PR China; 3 Department of Thoracic Surgery, First Affiliated Hospital of Nanjing Medical University, 300 Guangzhou Road, Nanjing 210029, PR China; 4 Department of Gastroenterology, First Affiliated Hospital of Nanjing Medical University, 300 Guangzhou Road, Nanjing 210029, PR China; 5 Department of Cardiology, First Affiliated Hospital of Nanjing Medical University, 300 Guangzhou Road, Nanjing 210029, PR China; 6 Department of Oncology, Clinical Medical College of Yangzhou University, No. 98 Nantong Western road, Yangzhou 225001, PR China; 7 State Key Laboratory of Natural Medicines and Department of Pharmacognosy, China Pharmaceutical University, No. 24 Tongjia Lane, Nanjing, 210009, China; 8 Department of Emergency, First Affiliated Hospital of Nanjing Medical University, 300 Guangzhou Road, Nanjing 210029, PR China; 9 State Key Laboratory of Analytical Chemistry for Life Science, School of Chemistry and Chemical Engineering, Nanjing University, No. 22 Hankou Road, Nanjing 210093, PR China; 10 Department of Oncology, Third Affiliated Hospital of Soochow University, 185 Juqian Road, Changzhou 213003, PR China; 11 Cancer Center of Nanjing Medical University, Nanjing 210029, China

## Abstract

The differential expression of microRNAs (miRNAs) in plasma of gastric cancer (GC) patients may serve as a diagnostic biomarker. A total of 33 miRNAs were identified through the initial screening phase (3 GC pools vs. 1 normal control (NC) pool) using quantitative reverse transcription polymerase chain reaction (qRT-PCR) based Exiqon panel (miRCURY-Ready-to-Use-PCR-Human-panel-I + II-V1.M). By qRT-PCR, these miRNAs were further assessed in training (30 GC VS. 30 NCs) and testing stages (71 GC VS. 61 NCs). We discovered a plasma miRNA signature including five up-regulated miRNAs (*miR-185*, *miR-20a*, *miR-210*, *miR-25* and *miR-92b*), and this signature was evaluated to be a potential diagnostic marker of GC. The areas under the receiver operating characteristic curve of the signature were 0.86, 0.74 and 0.87 for the training, testing and the external validation stages (32 GC VS. 18 NCs), respectively. The five miRNAs were consistently dysregulated in GC tissues (n = 30). Moreover, *miR-185* was decreased while *miR-20a*, *miR-210* and *miR-92b* were increased in arterial plasma (n = 38). However, none of the miRNAs in the exosomes showed different expression between 10 GC patients and 10 NCs. In conclusion, we identified a five-miRNA signature in the peripheral plasma which could serve as a non-invasive biomarker in detection of GC.

Gastric cancer (GC) is the fifth most common malignancy and the third leading cause of cancer-related death all around the world in 2012. Approximately 50% of cases occur in Eastern Asia (the majority of which occur in China)^1^. Most patients are diagnosed with middle or late stage disease, with 35% of patients demonstrating distant metastases and 90% having lymph node metastases^2^. Despite increased understanding of the molecular and clinical characteristics of GC^3^ as well as numerous advances in screening and treatment strategies^4^,^5^,^6^,^7^, the prognosis of GC is still poor. Therefore, many new research efforts have focused on early detection and intervention to increase the possibility of curable resections and thus prolong the survival of GC patients. In clinical practice, gastroscopic or surgical biopsy is used to diagnose GC. However, the approach is considered invasive and success may be limited by the experience of operators. Additionally, it is difficult to advocate for mass screening in susceptible populations because of the high cost of endoscopic procedures. Non-invasive markers such as carbohydrate antigen 19-9 (CA19-9) and carcinoembryonic antigen (CEA) have not adequately shown sufficient sensitivity and specificity to be of routine use in non-symptomatic patients^8^. Thus, novel and reliable biomarkers to diagnose GC for early intervention are urgently needed.

Recent research has demonstrated that circulating miRNAs that originate from tumors can be stably detected in peripheral blood and may aid in the detection and diagnosis of various types of cancer^9^. These findings have opened up the possibility of a new and promising era in the screening and monitoring of cancer patients. Specifically, many studies have explored the differential expression of circulating miRNAs in GC and identified some potential miRNA biomarkers for the detection^8^,^10^,^11^. Unfortunately, these results were not reproducible between laboratories, and these inconsistencies might be explained by differences in research methods and tested populations. At present, there is no consensus on suitable small RNA reference genes for clinical testing. *MiR-16* was used as a reference gene in some studies^12^,^13^. But the optimal way to normalize miRNA between body fluid samples (including those obtained from systemic circulation) is considered to be an absolute quantification procedure based on the spiked-in normalization method^14^,^15^,^16^.

In the current study, we performed plasma miRNA profiling through the quantitative reverse transcription polymerase chain reaction (qRT-PCR) based miRCURY platform^17^ followed by validation of absolute quantification based on qRT-PCR, and the expression profile of selected miRNAs was then assessed in the GC tissue. Peripheral plasma miRNAs were also compared to those obtained from arterial plasma samples. Peripheral plasma exosomal miRNAs were further analyzed to investigate the potential form of the miRNAs in the circulation that could be useful in the detection of gastric cancer.

## Results

### Characteristics of subjects

**Figure 1:**
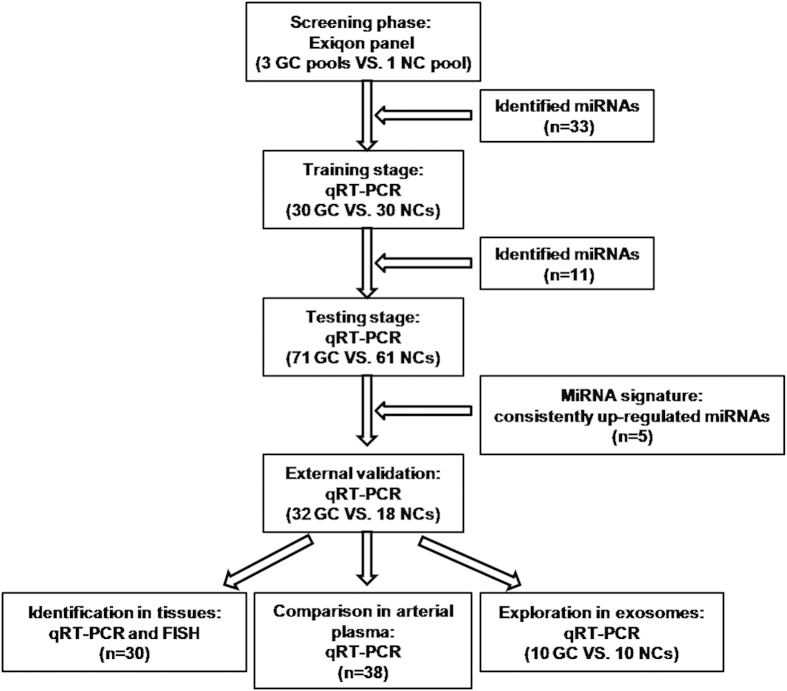
Overview of the experiment design. GC: gastric cancer; NC: normal control.

A total of 242 subjects, including 133 GC patients and 109 normal controls (NCs), were enrolled in our study to assess the differently expressed miRNAs in the peripheral plasma of GC patients. As shown in Table 1, the GC patients and NCs were divided into three stages: the training stage, the testing stage, and the external validation stage (The flow chart of the experiment was shown in Fig. 1). No significant difference in age or gender distribution was observed between patients and controls in any of the three cohorts (p-values > 0.05).

### MiRNAs profiling from pooled plasma samples

Based on the qRT-PCR platform, the Exiqon miRCURY-Ready-to-Use-PCR-Human-panel-I + II-V1.M was utilized to analyze 168 miRNAs that are expressed in plasma/serum. This method was used to identify differently expressed miRNAs between 3 peripheral plasma pools from 30 GC cases and 1 pooled sample from 10 controls. Each miRNA was assayed twice on 384-well plates by qRT-PCR and the miRNAs with cycle threshold (Ct) value less than 37 and 5 lower than negative control (No Template Control, NTC) in the panel were included in data analysis. Among the 168 miRNAs analyzed, the expression of 33 miRNAs (29 up-regulated miRNAs and 4 down-regulated miRNAs; Supplementary Table S1 online) was altered with at least a 1.5-fold change in all 3 pooled GC samples compared to the NC pool sample. These miRNAs were chosen to further validation in the experiments outlined below.

### Evaluation of miRNAs in peripheral plasma by qRT-PCR

**Figure 2:**
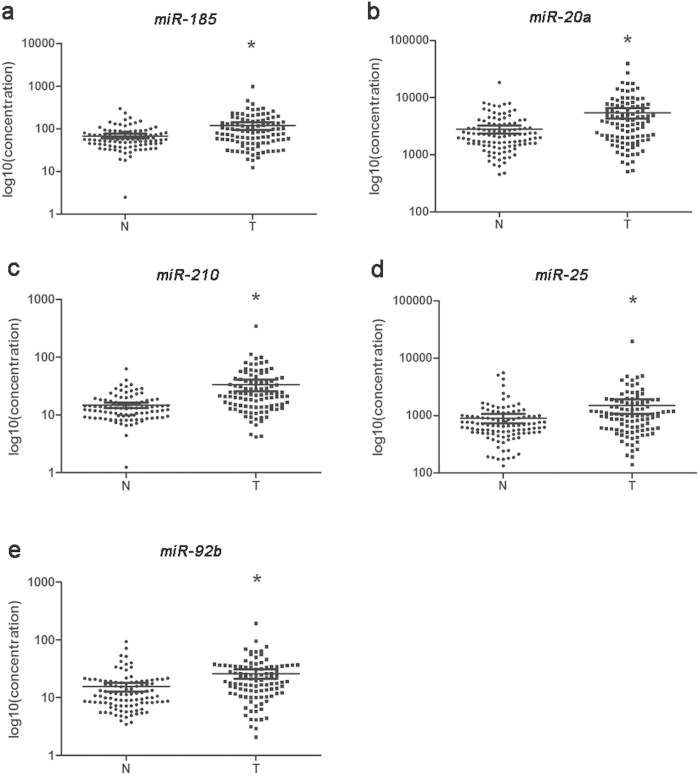
Expression levels of five miRNAs in the peripheral plasma of 101 GC patients and 91 controls (in the training and testing stages). Y axis was presented as log10 (concentration; fmol/L). **a**: *miR-185*; **b**: *miR-20a*; **c**: *miR-210*; **d**: *miR-25*; **e**: *miR-92b*; N: normal controls; T: tumor. Horizontal line: mean with 95% CI. *P-value < 0.05.

To obtain the absolute concentration of each miRNA identified through the screening phase in plasma of GC patients and NCs, the methods^18^ based on the standard curve of synthetic miRNAs were performed. A total of 11 miRNAs showed differential expression in the training stage and were subjected to validation in the testing phase. In the larger cohort, 5 of 11 miRNAs (*miR-185*, *miR-20a*, *miR-210*, *miR-25* and *miR-92b*) were consistent with those in the training stage (Table 2; the other miRNAs were shown in the Supplementary Table S2 and Table S3 online). When the results of two stages were combined, *miR-185*, *miR-20a*, *miR-210*, *miR-25* and *miR-92b* were significantly up-regulated in peripheral plasma of GC patients compared with NCs (Fig. 2).

### Diagnostic value of miRNAs in peripheral plasma

**Figure 3:**
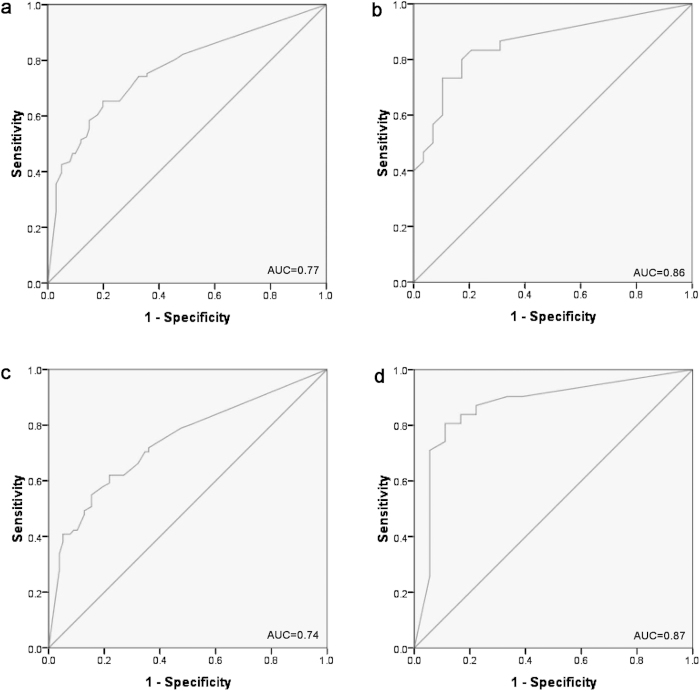
Receiver-operating characteristic (ROC) curve analyses of the five-miRNA signature to discriminate GC patients from normal controls. **a**: the combined two cohorts of training and testing stages (101 GC VS. 91 NCs); **b**: training stage (30 GC VS. 30 NCs); **c**: testing stage (71 GC VS. 61 NCs); **d**: external validation stage (32 GC VS. 18 NCs). GC: gastric cancer; NC: normal control. AUC: areas under the curve.

To evaluate the diagnostic value of the five identified miRNAs in discriminating GC from NCs, the data from training and testing stage were combined to calculate the optimal cutoff values for *miR-185*, *miR-20a*, *miR-210*, *miR-25* and *miR-92b* by using receiver operating characteristic (ROC) curves. The areas under the curve (AUC) were 0.65 (95% confidence interval (CI): 0.57–0.72), 0.67 (95% CI: 0.61–0.74), 0.75 (95% CI: 0.68–0.82), 0.65 (95% CI: 0.58–0.73) and 0.69 (95% CI: 0.62–0.76) for *miR-185*, *miR-20a*, *miR-210*, *miR-25* and *miR-92b*, respectively (Supplementary Figure S1 online). The risk score function (RSF) for each subject was calculated and used to explore the sensitivity and specificity of the five-miRNA signature. The signature showed a greater ability than any individual miRNA in detecting GC in the combined cohorts with AUC of 0.77 (95% CI: 0.70–0.84; Fig. 3a). The optimal cutoff value was indicated at 3.21, which demonstrated a sensitivity of 0.65 and a specificity of 0.8. The diagnostic value of the five-miRNA signature was also explored in the two stages separately and led to AUC of 0.86 (95% CI: 0.76–0.96; Fig. 3b) and 0.74 (95% CI: 0.66–0.82; Fig. 3c) in the training and testing stage, respectively. The sensitivity and specificity was 0.89 and 0.73 in the training stage and 0.62 and 0.78 for the testing stage when 3.21 was used as the cutoff value.

The external cohort included 32 GC and 18 NCs that were additionally analyzed to further determine the diagnostic capacity of the five-miRNA signature for diagnosing GC. Compared to NCs, all five miRNAs were found to be up-regulated in plasma samples from GC patients (Supplementary Table S4 online). As shown in Fig. 3d, the signature could accurately discriminate GC patients from normal individuals with AUC of 0.87 (95% CI: 0.76–0.98). And the sensitivity and specificity of the five-miRNA signature was 0.82 and 0.86.

The association of the five peripheral plasma miRNAs with clinical stage was also evaluated for all 133 GC patients. However, none of the five miRNAs showed significantly different expression in patients with stage III + IV compared to those with stage I + II. Furthermore, we also compared the levels of the five miRNAs in plasma from GC patients with metastasis to those without. Similarly, no significant difference of the five miRNAs was observed between the two cohorts (data not shown).

### Identification of miRNAs in tissue samples

**Figure 4:**
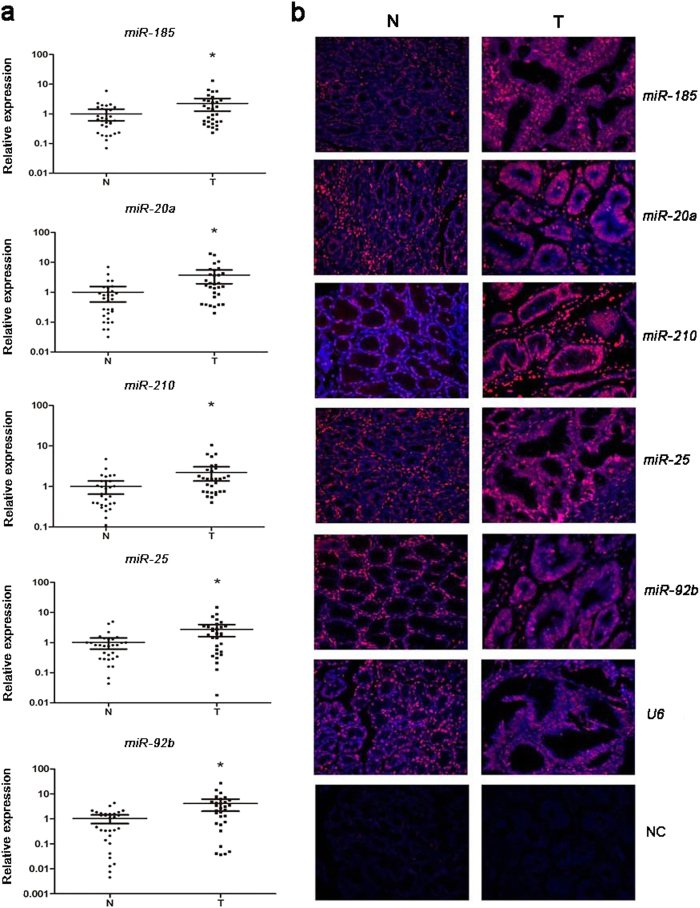
Expression of the five selected miRNAs in the tumor tissues of GC patients. *MiR-185*, *miR-20a*, *miR-210*, *miR-25* and *miR-92b* were significantly up-regulated in GC tissues by qRT-PCR (**a**: 30 pairs of tumor and matched normal tissues) and FISH (**b**: pictures were selected from 10 pairs of GC tissues and matched normal tissues). Y axis was presented as relative expression (normalized to *U6*; 2^−ΔΔCt^). Horizontal line: mean with 95% CI. *U6* was used as positive control. N: normal controls; T: tumor. NC: negative control. Red: Cy5-tyramide showing positive hybridization signals, blue: DAPI-stained nuclei. Original magnification 400×. *P-value < 0.05.

The expression levels of miRNAs identified were examined in an additional of thirty pairs of tissue samples to explore the association of the five miRNAs in peripheral plasma and tissue of GC patients. As shown in Fig. 4a, the expression of *miR-185*, *miR-20a*, *miR-210*, *miR-25* and *miR-92b* was significantly higher in tumor than in normal tissues, and this correlation was consistent with results obtained from peripheral plasma. Fluorescence *in situ* hybridization (FISH) was conducted in an area containing at least 85% tumor in order to appropriately visualize the expression of *miR-185*, *miR-20a*, *miR-210*, *miR-25* and *miR-92b* in FFPE tissues of GC patients (Fig. 4b), and these experiments provided consistent results concerning the miRNA levels that the five miRNAs were up-regulated in GC patients.

### Comparison of miRNAs in peripheral and arterial plasma

**Figure 5:**
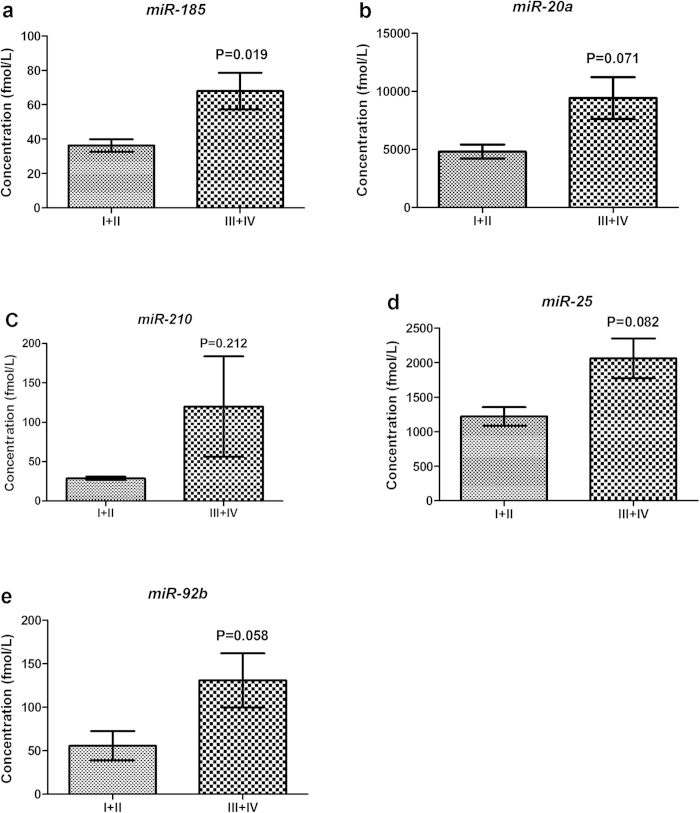
Comparison of the five miRNAs in arterial plasma of GC patients diagnosed with early (I + II; n = 14) and late (III + IV; n = 24) stage disease. *MiR-185* was significantly up-regulated in late stage compared to early stage patients. **a**: *miR-185*; **b**: *miR-20a*; **c**: *miR-210*; **d**: *miR-25*; **e**: *miR-92b*. Error bar: standard error.

To determine whether there was any difference in miRNA expression between peripheral and arterial plasma, the absolute concentration of *miR-185*, *miR-20a*, *miR-210*, *miR-25* and *miR-92b* in another 38 GC arterial plasma samples was evaluated and compared with those in 133 peripheral plasma samples from GC patients. Interestingly, in arterial plasma, *miR-20a*, *miR-210* and *miR-92b* were significantly up-regulated while *miR-185* was significantly decreased with a fold-change of 1.37, 2.35, 3.34 and 0.44, respectively (Supplementary Table S5 online). When compared the expression levels of the five miRNAs in arterial plasma from patients with different stages (4 with I, 10 with II, 20 with III and 4 with IV), all five miRNAs were found to be elevated but only *miR-185* was significantly up-regulated in patients with stage III + IV compared to those with stage I + II (Fig. 5).

### Exploration of miRNAs in peripheral plasma exosomes

To investigate the potential form of the identified miRNAs in peripheral plasma of GC patients, exosomal miRNAs were examined in ten peripheral plasma samples from GC patients and compared to ten NC samples. None of the five miRNAs (*miR-185*, *miR-20a*, *miR-210*, *miR-25* and *miR-92b*) from GC patients demonstrated a significantly different expression level in peripheral plasma exosomes as compared to controls (Supplementary Table S6 online).

### **Bioinformatics analysis of** ***miR-185***, ***miR-20a***, ***miR-210***, ***miR-25*** **and** ***miR-92b***

The putative target genes of the miRNAs were identified by miRanda, miRDB, miRWalk, RNA22, and Targetscan. These programs were used for KEGG pathway analysis to investigate the pathways which were significantly associated with the five identified miRNAs (Supplementary Table S7 online). Among the five miRNAs, *miR-20a* seemed to be much more related to pathological mechanisms of cancer such as by interacting with MAPK and mTOR signaling.

## Discussion

In this study, we identified five miRNAs which were up-regulated in the peripheral plasma of GC patients compared to controls. The diagnostic role of circulating miRNAs in GC has been reported in some previous studies. In addition to population and sample diversity, differences in analytic methods may have also contributed to inconsistent results between studies. Compared to TaqMan platform that was used in the study of Zhu *et al*.^19^, we utilized Exiqon miRNA qPCR panels which appeared to show better sensitivity and linearity with measurements of miRNAs in relatively low abundance. This platform was used specifically in the screening phase to conduct peripheral plasma miRNA profiling^17^. In the following validation stages, the expression level of each miRNA in plasma of GC patients and NCs were identified by the method of absolute concentration analysis, which might present better information than the relative quantification methods that have been applied in most previous studies^14^. By the relative quantification method, Zhu^19^ identified plasma *miR-16* as a potential diagnostic marker for GC. However, *miR-16* was usually used as a reference molecule in the circulation and could also be secreted from and influenced by haemolysis^12^,^20^,^21^. In the training and testing phases of our experiment, *miR-185*, *miR-20a*, *miR-210*, *miR-25* and *miR-92b* were found to be significantly up-regulated in GC. The five-miRNA signature could accurately discriminate patients from normal individuals. However, some promising miRNAs demonstrated in previous studies^22^,^23^ (such as *miR-21*) was identified in the screening phase but was not applicable in the following stages of the experiment. The external validation stage further demonstrated the reliability of the diagnostic value of the five-miRNA signature. Of note, consistent miRNA expression in GC tissue samples suggested the important roles for these miRNAs in tumorigenesis and progression of the disease.

Among the five up-regulated miRNAs, *miR-185* seemed to play a controversial role in the development and progression of GC, suggesting its conflicting function in GC^24^,^25^. Unlike *miR-185*, circulating *miR-20a* was reported to be a noninvasive biomarker of GC, and this finding was consistently demonstrated in three recent studies^8^,^26^,^27^. It was suggested that elevated level of circulating *miR-20a* could promote growth, migration and invasion of GC cells and enhance the chemoresistance of GC cells to cisplatin and docetaxel^28^,^29^. As a member of the *miR-17-92* cluster, *miR-20a* could also sustain the self-renewal function of GC stem cells and promote the proliferation of GC cells by targeting E2F transcription factor 1 (*E2F1*)^30^. Thus, *miR-20a* might serve as an oncogene in GC. *MiR-210*, as a typical hypoxia-related miRNA, was reportedly induced by a hypoxia inducible transcription factor (HIF) dependent mechanism^31^,^32^. Recently, serum *miR-210* was reported to be one of four serum miRNAs (*miR-103*, *miR-107*, *miR-194* and *miR-210*) which could serve as biomarkers for the early detection of diffuse-type GC^33^. Meanwhile, based on The Cancer Genome Atlas (TCGA) data set, *miR-210* was identified as a member of the pan-cancer oncogenic miRNA superfamily^34^. The mechanism of *miR-210* in GC has not been explicitly reported, but it may promote growth and metastasis in various other cancer types, serving as a negative prognostic factor^35^. As for *miR-25* and *miR-92b*, Zhu *et al*.^10^ revealed that plasma *miR-25* might be a potential biomarker in the detection of GC through relative quantification. Additionally, Omura *et al*.^36^ reported that high *miR-92b* level could be used to predict relapse of GC after S-1 adjuvant chemotherapy. In our study, the five-miRNA signature was closely associated with a diagnosis of GC. Further research of these miRNAs in GC formation and development is warranted.

The diagnostic value of the circulating miRNAs identified in our study was also evaluated in some other cancers. Up-regulation of circulating *miR-20a* could be used to facilitate the diagnosis of nasopharyngeal carcinoma^37^ as well as astrocytoma^38^. High level of circulating *miR-210* was found in pancreatic ductal adenocarcinoma^39^, clear cell renal cell carcinoma^40^ and adrenocortical tumors^41^. Elevated plasma *miR-25* was identified as a diagnostic and monitoring biomarker in esophageal squamous cell carcinoma^42^. These suggested that the specificity of circulating miRNAs as diagnostic markers is worthy further study.

Circulating miRNAs were believed to be passively leaked or actively transported from cells during tumorigenesis and packaged into small membranous vesicles or protected by the formation of a protein-miRNA complex^43^,^44^,^45^. In the present study, we also assessed the expression of the five plasma miRNAs in the tissues by RT-PCR and FISH and found that all the five miRNAs were consistently up-regulated in the tissue samples of GC patients. The findings were consistent with and may verify the theory. As blood flows from arterial to venous circulation, we hypothesized that the expression level of circulating miRNAs secreted from tissues might be greater in the arterial plasma than those in the peripheral venous plasma. Interestingly, *miR-20a*, *miR-210*, and *miR-92b* were significantly up-regulated in the arterial circulation which might suggest that circulating *miR-20a*, *miR-210*, and *miR-92b* could be absorbed and transmitted to distant body sites and participate in biological processes (including tumorigenesis, metastasis and other pathways). The relatively stable concentration of *miR-25* (shown in Supplementary Table S5 online) in the arterial and peripheral venous plasma could reflect a relative balance of absorption and secretion of this particular miRNA in the circulation. On the other hand, *miR-185* demonstrated a significant reduction in arterial plasma than that in peripheral venous plasma. And arterial *miR-185* was the only miRNA that was found to be significantly associated with TNM stage. The phenomenon was interesting, fascinating and difficult to explain. We assumed that circulating *miR-185* released by tumor might stimulate the further secretion of *miR-185* according to some mechanism. However, the exact mechanism is needed to be further studied in the future. We further explored exosomal miRNAs from peripheral venous plasma to identify the potential form of the five miRNAs in circulation. However, no difference of them in peripheral plasma exosomes between GC patients and NCs was found. A representative study to characterize the extracellular form of circulating miRNA complexes in human plasma and serum recently revealed that the majority of miRNAs were co-purified with the Ago2 ribonucleoprotein complex but a minority of specific miRNAs (such as *miR-1979* and *miR-940*) associated predominantly with vesicles like exosomes. Their results showed that the four miRNAs in our study (*miR-25*, *miR-20a*, *miR-185* and *miR-210*) were co-purified with the Ago2 ribonucleoprotein complex other than exosomes in plasma while the form of *miR-92b* was undefined in the circulation. Our findings indirectly validated their results.

In conclusion, we identified a five-miRNA signature in the plasma of GC patients which could serve as a non-invasive biomarker in the detection of GC, indicating its potential application of plasma miRNAs in the screening of GC patients to improve clinical outcomes.

## Methods

### Study design, patients and samples

A total of 133 histopathologically confirmed GC patients and 109 healthy donors were recruited at First Affiliated Hospital of Nanjing Medical University between 2011 and 2013. In the initial screening phase (Fig. 1), 30 peripheral plasma samples from GC patients and 10 from NCs were randomly selected and pooled as 3 GC samples and 1 NC sample (10 samples were pooled as 1 pool sample). According to the manufacturer’s protocol, total of 25 ng RNA extracted from each pooled sample was then reverse transcribed using the miRCURY Locked Nucleic Acid (LNA™) Universal Reverse Transcription (RT) microRNA PCR, Polyadenylation and cDNA synthesis kit (Exiqon miRNA qPCR panel, Vedbaek, Denmark) and was further subjected to Exiqon miRCURY-Ready-to-Use PCR-Human-panel-I + II-V1.M (Exiqon miRNA qPCR panel, Vedbaek, Denmark) which could detect 168 miRNAs in plasma/serum to identify differently expressed miRNAs on 7900HT real-time PCR system (Applied Biosystems, Foster City, CA, USA). Assays detected with 5 Ct’s less than the negative control (No Template Control, NTC), and with Ct <37 were included in the data analysis. An RNA spike-in (UniSp6) and a DNA spike-in (Sp3) were applied in the panel for quality controls to check if the technical performance of all samples is similar. All assays were inspected for appropriate melting curves and the melting temperature (Tm) was checked to be within known specifications. Normalization was performed based on the average of the normalizer assays in the panel which included *miR-191-5p*, *miR-423-5p*, *miR-425-5p* and *miR-93-5p*. And normalized Ct (ΔCt) = average Ct (assay) – average Ct (normalizer assays). The relative expression between GC patients and NCs was assessed by 2^−ΔΔCt^ method.

In the training stage, 30 GC samples and 30 NCs were used to confirm the miRNAs discovered through screening phase by qRT-PCR. In the testing phase, the miRNAs identified in the training stage were further validated in plasma samples of 71 GC patients and 61 NCs. An external cohort including 32 cases and 18 controls were analyzed to assess the diagnostic value of the five-miRNA signature in GC. An additional of thirty pairs of GC tissue specimens and matched normal tissues were obtained from surgery patients and used to verify the expression level of identified miRNAs. Arterial blood samples from another 38 GC patients were collected to compare the difference of miRNAs between peripheral and arterial plasma. And exosomal miRNAs from ten patients and NCs were further assessed to explore the potential form of the miRNAs in the peripheral plasma.

Blood samples of healthy controls and GC patients before initial treatment were collected by using ethylenediaminetetraacetic acid (EDTA)-containing tubes. Cell-free plasma was isolated from blood samples within 12 hours after collection using a two-step protocol (1,500 r.p.m. for 10 min, 12,000 r.p.m. for 2 min) to prevent contamination by cellular nucleic acids and then stored at −80 °C until further processing. Tissue specimens were obtained from surgery patients and kept in liquid nitrogen.

The study was approved by Institutional Review Boards of the First Affiliated Hospital of Nanjing Medical University and informed consent was taken from each participant. And the study was carried out in accordance with the approved guidelines by the Hospital Ethics Committee.

### Isolation of Exosomes

Exosomes were extracted from peripheral plasma using ExoQuick Exosome Precipitation Solution (System Biosciences, Mountain View, Calif) according to the manufacturer’s instructions. Precipitated from 100 μl ExoQuick exosome precipitation solutions and 400 μl plasma, exosome pellets were lysed in 200 μl RNase-free water for further analysis.

### RNA extraction

Total RNA from 200 μl plasma or exosomes was extracted using the mirVana PARIS Kit (Ambion, Austin, TX, USA) according to the protocol. For normalization of the sample-to-sample variation, 5 μl of synthetic C.elegans *miR-39* (5 nM/L, RiboBio, Guangzhou, China) was added to each sample after the addition of denaturing solution (Ambion, Austin, TX, USA). For tissue samples, total RNA was isolated using Trizol (Invitrogen, Carlsbad, CA, USA) according to the manufacturer’s instructions. Total RNA was eluted in 100 μl of RNase-free water and stored at −80 °C until analysis. The concentration and purity of the total RNA was evaluated by using the ultraviolet spectrophotometer. For plasma samples, total RNA with concentration <10 ng/ul was not included in the analysis. And the mean and standard deviation of concentration of plasma RNA was 31.23 ± 12.36 ng/ul (range: 15.73~48.62 ng/ul). Thus, no carrier molecule was added during RNA isolation.

### Quantitative reverse transcription polymerase chain reaction (qRT-PCR)

The specific primers of reverse transcription (RT) and polymerase chain reaction (PCR) from Bulge-Loop™ miRNA qRT-PCR Primer Set (RiboBio, Guangzhou, China) were applied to amplify miRNAs. The amount of PCR product was detected by evaluating the level of fluorescence emitted by SYBR Green (SYBR® Premix Ex Taq™ II, TaKaRa, Dalian, China). RT and PCR were performed as previously described^18^,^46^. RT reactions were performed at 42 °C for 60 min followed by 70 °C for 10 min. The qRT-PCR was conducted on 7900HT real-time PCR system (Applied Biosystems, Foster City, CA, USA) at 95 °C for 20 sec, followed by 40 cycles of 95 °C for 10 sec, 60 °C for 20 sec and then 70 °C for 10 sec. The melting analysis was added finally to evaluate the specificity of PCR products.

The amount of miRNA in plasma samples was calculated based on a standard curve constructed with the use of synthetic miRNAs (micrON miRNA mimic, RiboBio, Guangzhou, China)^18^. The expression of miRNAs from tissue samples and exosomes was determined using the 2^−ΔΔCt^ method relative to *RNU6B* (*U6*) and *cel-miR-39*^47^.

### Fluorescence ****in situ**** hybridization (FISH)

Antisense LNA-modified oligodeoxynucleotide probes were purchased from RiboBio (Guangzhou, China). Ten tissue specimens of GC and matched normal tissues were fixed in 10% neutral buffered formalin for 24 hours and passed through dehydration, clearing, and paraffin-embedding steps. Approximately 5 μm thick sections from tissue blocks were de-paraffinized, washed, air-dried, and subjected to antigen retrieval in 0.1 mmol/L of citric acid. Subsequently, slides were rinsed, washed and subjected to a pre-hybridization procedure (3% bovine serum albumin (BSA) in 4 × saline sodium citrate (SSC), 25 °C for 20 min) to block nonspecific binding of probes and minimize background signals. Hybridizations were then performed in hybridization buffer (10% dextran sulfate in 4 × SSC, 6000 μg/ml miRNA probe) in dark at 25 °C for 60 min. Slides were then washed in 2 × SSC with 0.5% Tween-20 three times for 5 min respectively at room temperature and immersed in phosphate buffered saline (PBS) solution followed by nuclear staining with 4’,6-diamidino-2-phenylindole (DAPI). Hybridizations were observed with WIB filters under fluorescence microscope Olympus AX-70.

### Statistical analysis

Mann–Whitney test was used to compare the difference of miRNA concentration in different groups. Demographic and clinical characteristics among groups and their relationship with miRNA were compared using one-way ANOVA or χ^2^ test. A formula for calculating risk score of miRNA for GC was developed^8^. The score (*S*) of each miRNA was set as 1 if the expression level of the miRNA was greater than the optimal cutoff value of corresponding miRNA level in controls, otherwise as 0. The risk score function (RSF) for patient *i* was calculated by: 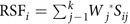


Here, the score (S_*ij*_) of miRNA *j* on patient *i* was weighted by W_*j*_, the regression coefficient estimated by univariate logistic regression models for each miRNA. The receiver operating characteristic (ROC) curves were used to estimate the diagnostic value of the identified miRNAs on GC.

All statistical analysis was performed using SPSS15.0 software (SPSS Inc., Chicago, IL, USA). A P-value <0.05 was considered to indicate a statistically significant difference.

## Additional Information

**How to cite this article**: Zhou, X. *et al*. Diagnostic value of a plasma microRNA signature in gastric cancer: a microRNA expression analysis. *Sci. Rep*. **5**, 11251; doi: 10.1038/srep11251 (2015).

## Supplementary Material

Supplementary Information

## Figures and Tables

**Table 1:** Characteristics of 133 GC patients and 109 normal controls enrolled in the study.

	**Training stage (n = 60)**		**Testing stage (n = 132)**		**External validation stage (n = 50)**	
Variables	Cases (%)	Controls (%)	Cases (%)	Controls (%)	Cases (%)	Controls (%)
Number	30	30	71	61	32	18
**Gender**						
Male	20 (66.7)	18 (60)	42 (59.1)	37 (60.7)	21 (65.6)	11 (61.1)
Female	10 (33.3)	12 (40)	29 (40.9)	24 (39.3)	11 (34.4)	7 (38.9)
**Age**						
<60	13 (43.3)	12 (40)	33 (46.5)	26 (42.7)	15 (46.9)	8 (44.4)
≥60	17 (56.7)	18 (60)	38 (53.5)	35 (57.3)	17 (53.1)	10 (55.6)
**Location**						
Proximal	7 (23.3)		20 (28.2)		11 (34.4)	
Middle	12 (40)		33 (46.5)		12 (37.5)	
Distal	11 (36.7)		18 (25.3)		9 (28.1)	
**Differentiation**						
Well	6 (20)		19 (26.8)		8 (25)	
Moderately	11 (36.7)		28 (39.4)		13 (40.6)	
Poorly	13 (43.3)		24 (33.8)		11 (34.4)	
**T classification**						
T1	4 (13.3)		16 (22.5)		8 (25)	
T2	4 (13.3)		14 (19.7)		4 (12.5)	
T3	5 (16.7)		15 (21.1)		7 (21.9)	
T4	17 (56.7)		26 (36.7)		13 (40.6)	
**N classification**						
N0	5 (16.7)		17 (23.9)		9 (28.1)	
N1	8 (26.7)		18 (25.4)		7 (21.9)	
N2	10 (33.3)		19 (26.8)		5 (15.6)	
N3	7 (23.3)		17 (23.9)		11 (34.4)	
**Metastasis**						
M0	27 (90)		62 (87.3)		28 (87.5)	
M1	3 (10)		9 (12.7)		4 (12.5)	
**TNM stage**						
I	8 (26.7)		13 (18.3)		6 (18.8)	
II	5 (16.7)		14 (19.7)		5 (15.6)	
III	14 (46.6)		35 (49.3)		17 (53.1)	
IV	3 (10)		9 (12.7)		4 (12.5)	

**Table 2:** Expression levels of the five miRNAs in the peripheral plasma in the training and testing stages (presented as mean ± SD; fmol/L).

miRNA	Training stage				Testing stage				Combined	
	Controls	Cases	FC	P value	Controls	Cases	FC	P value	FC	P value
*miR-185*	80.8 ± 40.1	138 ± 54.8	1.70	0.006	74 ± 34.1	122 ± 92.1	1.65	0.016	1.82	<0.001
*miR-20a*	3248 ± 1860	5880 ± 2153	1.81	<0.001	3098 ± 1801	5816 ± 4893	1.88	0.025	2.05	<0.001
*miR-210*	14.8 ± 4.54	35.5 ± 17.9	2.39	<0.001	15.5 ± 6.1	35.4 ± 26.4	2.28	<0.001	2.39	<0.001
*miR-25*	590 ± 317	980 ± 382	1.66	0.001	1011 ± 612	1719 ± 1218	1.70	0.002	1.70	<0.001
*miR-92b*	18.6 ± 7.6	35.5 ± 16.7	1.90	0.006	14.1 ± 7	24.2 ± 14.7	1.71	<0.001	1.74	<0.001

FC: fold change.

## References

[b1] Ferlay J. . Cancer incidence and mortality worldwide: Sources, methods and major patterns in GLOBOCAN 2012. Int J Cancer. 136, E359–386; 10.1002/ijc.29210 (2015). 25220842

[b2] Jackson C., Cunningham D. & Oliveira J. Gastric cancer: ESMO clinical recommendations for diagnosis, treatment and follow-up. Ann Oncol. 20 Suppl 4, 34–36; 10.1093/annonc/mdp122 (2009). 19454457

[b3] Comprehensive molecular characterization of gastric adenocarcinoma. Nature. 10.1038/nature13480 (2014). PMC4170219 25079317

[b4] Paoletti X. . Benefit of adjuvant chemotherapy for resectable gastric cancer: a meta-analysis. JAMA. 303, 1729–1737; 10.1001/jama.2010.534 (2010). 20442389

[b5] Waddell T. . Epirubicin, oxaliplatin, and capecitabine with or without panitumumab for patients with previously untreated advanced oesophagogastric cancer (REAL3): a randomised, open-label phase 3 trial. Lancet Oncol. 14, 481–489; 10.1016/S1470-2045(13)70096-2 (2013). 23594787 PMC3669518

[b6] Cunningham D. . Perioperative chemotherapy versus surgery alone for resectable gastroesophageal cancer. N Engl J Med. 355, 11–20; 10.1056/NEJMoa055531 (2006). 16822992

[b7] Ychou M. . Perioperative chemotherapy compared with surgery alone for resectable gastroesophageal adenocarcinoma: an FNCLCC and FFCD multicenter phase III trial. J Clin Oncol. 29, 1715–1721; doi: 10.1200/JCO.2010.33.0597 (2011). 21444866

[b8] Liu R. . A five-microRNA signature identified from genome-wide serum microRNA expression profiling serves as a fingerprint for gastric cancer diagnosis. Eur J Cancer. 47, 784–791; 10.1016/j.ejca.2010.10.025 (2011). 21112772

[b9] Mitchell P. S. . Circulating microRNAs as stable blood-based markers for cancer detection. Proc Natl Acad Sci U S A. 105, 10513–10518; 10.1073/pnas.0804549105 (2008). 18663219 PMC2492472

[b10] Zhu C. . A five-microRNA panel in plasma was identified as potential biomarker for early detection of gastric cancer. Br J Cancer. 10.1038/bjc.2014.119 (2014). PMC4007222 24595006

[b11] Song M. Y. . Identification of serum microRNAs as novel non-invasive biomarkers for early detection of gastric cancer. PLoS One. 7, e33608; 10.1371/journal.pone.0033608 (2012). 22432036 PMC3303856

[b12] Song J. . Identification of suitable reference genes for qPCR analysis of serum microRNA in gastric cancer patients. Dig Dis Sci. 57, 897–904; 10.1007/s10620-011-1981-7 (2012). 22198701

[b13] Xiang M. . U6 is not a suitable endogenous control for the quantification of circulating microRNAs. Biochem Biophys Res Commun. 454, 210–214; 10.1016/j.bbrc.2014.10.064 (2014). 25450382

[b14] Sellars M. J. . Real-time RT-PCR quantification of Kuruma shrimp transcripts: a comparison of relative and absolute quantification procedures. J Biotechnol. 129, 391–399; 10.1016/j.jbiotec.2007.01.029 (2007). 17350129

[b15] Kroh E. M., Parkin R. K., Mitchell P. S. & Tewari M. Analysis of circulating microRNA biomarkers in plasma and serum using quantitative reverse transcription-PCR (qRT-PCR). Methods. 50, 298–301; 10.1016/j.ymeth.2010.01.032 (2010). 20146939 PMC4186708

[b16] Zhou X. . Prognostic value of miR-21 in various cancers: an updating meta-analysis. PLoS One. 9, e102413; 10.1371/journal.pone.0102413 (2014). 25019505 PMC4097394

[b17] Jensen S. G. . Evaluation of two commercial global miRNA expression profiling platforms for detection of less abundant miRNAs. BMC Genomics. 12, 435; 10.1186/1471-2164-12-435 (2011). 21867561 PMC3184117

[b18] Zhao D. S. . Serum miR-210 and miR-30a expressions tend to revert to fetal levels in Chinese adult patients with chronic heart failure. Cardiovasc Pathol. 22, 444–450; 10.1016/j.carpath.2013.04.001 (2013). 23660476

[b19] Zhu C. . A five-microRNA panel in plasma was identified as potential biomarker for early detection of gastric cancer. Br J Cancer. 110, 2291–2299; 10.1038/bjc.2014.119 (2014). 24595006 PMC4007222

[b20] Shiotani A. . Identification of serum miRNAs as novel non-invasive biomarkers for detection of high risk for early gastric cancer. Br J Cancer. 109, 2323–2330; 10.1038/bjc.2013.596 (2013). 24104965 PMC3817334

[b21] Kirschner M. B. . Haemolysis during sample preparation alters microRNA content of plasma. PLoS One. 6, e24145; 10.1371/journal.pone.0024145 (2011). 21909417 PMC3164711

[b22] Li B. S. . Plasma microRNAs, miR-223, miR-21 and miR-218, as novel potential biomarkers for gastric cancer detection. PLoS One. 7, e41629; 10.1371/journal.pone.0041629 (2012). 22860003 PMC3408505

[b23] Wang B. & Zhang Q. The expression and clinical significance of circulating microRNA-21 in serum of five solid tumors. J Cancer Res Clin Oncol. 138, 1659–1666; 10.1007/s00432-012-1244-9 (2012). 22638884 PMC11824721

[b24] Yao Y. . MicroRNA profiling of human gastric cancer. Mol Med Rep. 2, 963–970; 10.3892/mmr_00000199 (2009). 21475928

[b25] Tan Z. . miR-185 is an independent prognosis factor and suppresses tumor metastasis in gastric cancer. Mol Cell Biochem. 386, 223–231; 10.1007/s11010-013-1860-y (2014). 24352663

[b26] Wang M. . Circulating miR-17-5p and miR-20a: molecular markers for gastric cancer. Mol Med Rep. 5, 1514–1520; 10.3892/mmr.2012.828 (2012). 22406928

[b27] Cai H. . Plasma microRNAs serve as novel potential biomarkers for early detection of gastric cancer. Med Oncol. 30, 452; 10.1007/s12032-012-0452-0 (2013). 23307259

[b28] Kim B. H., Hong S. W., Kim A., Choi S. H. & Yoon S. O. Prognostic implications for high expression of oncogenic microRNAs in advanced gastric carcinoma. J Surg Oncol. 107, 505–510; 10.1002/jso.23271 (2013). 22996433

[b29] Li X. . Involvement of miR-20a in promoting gastric cancer progression by targeting early growth response 2 (EGR2). Int J Mol Sci. 14, 16226–16239; 10.3390/ijms140816226 (2013). 23924943 PMC3759908

[b30] Wu Q. . MiR-19b/20a/92a regulates the self-renewal and proliferation of gastric cancer stem cells. J Cell Sci. 126, 4220–4229; 10.1242/jcs.127944 (2013). 23868977

[b31] Kulshreshtha R. . A microRNA signature of hypoxia. Mol Cell Biol. 27, 1859–1867; doi: 10.1128/MCB.01395-06 (2007). 17194750 PMC1820461

[b32] Chan Y. C., Banerjee J., Choi S. Y. & Sen C. K. miR-210: the master hypoxamir. Microcirculation. 19, 215–223; 10.1111/j.1549-8719.2011.00154.x (2012). 22171547 PMC3399423

[b33] Rotkrua P. . Circulating microRNAs as biomarkers for early detection of diffuse-type gastric cancer using a mouse model. Br J Cancer. 108, 932–940; 10.1038/bjc.2013.30 (2013). 23385731 PMC3590667

[b34] Hamilton M. P. . Identification of a pan-cancer oncogenic microRNA superfamily anchored by a central core seed motif. Nat Commun. 4, 2730; 10.1038/ncomms3730 (2013). 24220575 PMC3868236

[b35] Wang J. . Elevated Expression of miR-210 Predicts Poor Survival of Cancer Patients: A Systematic Review and Meta-Analysis. PLoS One. 9, e89223; 10.1371/journal.pone.0089223 (2014). 24586608 PMC3930667

[b36] Omura T. . Relapse-associated microRNA in gastric cancer patients after S-1 adjuvant chemotherapy. Oncol Rep. 31, 613–618; 10.3892/or.2013.2900 (2014). 24317477

[b37] Zeng X. . Circulating miR-17, miR-20a, miR-29c, and miR-223 combined as non-invasive biomarkers in nasopharyngeal carcinoma. PLoS One. 7, e46367; 10.1371/journal.pone.0046367 (2012). 23056289 PMC3466268

[b38] Zhi F. . Identification of 9 serum microRNAs as potential noninvasive biomarkers of human astrocytoma. Neuro Oncol. 10.1093/neuonc/nou169 (2014). PMC4483096 25140035

[b39] Wang J. . MicroRNAs in plasma of pancreatic ductal adenocarcinoma patients as novel blood-based biomarkers of disease. Cancer Prev Res (Phila). 2, 807–813; 10.1158/1940-6207.CAPR-09-0094 (2009). 19723895 PMC5859193

[b40] Zhao A., Li G., Peoc’h M., Genin C. & Gigante M. Serum miR-210 as a novel biomarker for molecular diagnosis of clear cell renal cell carcinoma. Exp Mol Pathol. 94, 115–120; 10.1016/j.yexmp.2012.10.005 (2013). 23064048

[b41] Szabo D. R. . Analysis of circulating microRNAs in adrenocortical tumors. Lab Invest. 94, 331–339; 10.1038/labinvest.2013.148 (2014). 24336071

[b42] Komatsu S. . Plasma microRNA profiles: identification of miR-25 as a novel diagnostic and monitoring biomarker in oesophageal squamous cell carcinoma. Br J Cancer. 10.1038/bjc.2014.451 (2014). PMC4200091 25117812

[b43] Chen X. . Characterization of microRNAs in serum: a novel class of biomarkers for diagnosis of cancer and other diseases. Cell Res. 18, 997–1006; 10.1038/cr.2008.282 (2008). 18766170

[b44] Kosaka N. . Secretory mechanisms and intercellular transfer of microRNAs in living cells. J Biol Chem. 285, 17442–17452; 10.1074/jbc.M110.107821 (2010). 20353945 PMC2878508

[b45] Arroyo J. D. . Argonaute2 complexes carry a population of circulating microRNAs independent of vesicles in human plasma. Proc Natl Acad Sci U S A. 108, 5003–5008; 10.1073/pnas.1019055108 (2011). 21383194 PMC3064324

[b46] Qiu T. . MiR-145, miR-133a and miR-133b inhibit proliferation, migration, invasion and cell cycle progression via targeting transcription factor Sp1 in gastric cancer. FEBS Lett. 10.1016/j.febslet.2014.02.054 (2014). 24613927

[b47] Livak K. J. & Schmittgen T. D. Analysis of relative gene expression data using real-time quantitative PCR and the 2(-Delta Delta C(T)) Method. Methods. 25, 402–408; 10.1006/meth.2001.1262 (2001). 11846609

