# Supplementary material for: Diagnostic value of a plasma microRNA signature in gastric cancer: a microRNA expression analysis
Source: Sci Rep. 2015 Jun 10;5:11251. doi: 10.1038/srep11251 (PMC4462022; doi:10.1038/srep11251)

Diagnostic value of a plasma microRNA signature in gastric cancer: a microRNA expression analysis

Authors: Xin Zhou[1]; Wei Zhu[1] *; Hai Li[2]; Wei Wen[3]; Wenfang Cheng[4]; Fang Wang[5]; Yinxia Wu[6]; Lianwen Qi[7]; Yong Fan[7]; Yan Chen [8]; Yin Ding[9] ; Jing Xu[1] ; Jiaqi Qian[1] ; Zebo Huang[1]; Tongshan Wang[1]; Danxia Zhu[10]; Yongqian Shu[1,11] * ; Ping Liu[1,11] *

Institution: 1.Department of Oncology, First Affiliated Hospital of Nanjing Medical University, 300 Guangzhou Road, Nanjing 210029, PR China;
2.Department of Pathology, First Affiliated Hospital of Nanjing Medical University, 300 Guangzhou Road, Nanjing 210029, PR China;
3.Department of Thoracic Surgery, First Affiliated Hospital of Nanjing Medical University, 300 Guangzhou Road, Nanjing 210029, PR China;
4.Department of Gastroenterology, First Affiliated Hospital of Nanjing Medical University, 300 Guangzhou Road, Nanjing 210029, PR China;
5.Department of Cardiology, First Affiliated Hospital of Nanjing Medical University, 300 Guangzhou Road, Nanjing 210029, PR China;
6.Department of Oncology, Clinical Medical College of Yangzhou University, No. 98 Nantong Western road, Yangzhou 225001, PR China;
7.State Key Laboratory of Natural Medicines and Department of Pharmacognosy, China Pharmaceutical University, No. 24 Tongjia Lane, Nanjing, 210009,China;
8.Department of Emergency, First Affiliated Hospital of Nanjing Medical University, 300 Guangzhou Road, Nanjing 210029, PR China;
9.State Key Laboratory of Analytical Chemistry for Life Science, School of Chemistry and Chemical Engineering, Nanjing University, No. 22 Hankou Road, Nanjing 210093, PR China;
10.Department of Oncology, Third Affiliated Hospital of Soochow University, 185 Juqian Road, Changzhou 213003, PR China
11.Cancer Center of Nanjing Medical University, Nanjing 210029, China.
*Corresponding author: Dr. Wei Zhu, Prof. Yongqian Shu and Prof. Ping Liu.
Email address: zhuwei@njmu.edu.cn, shuyongqian@njmu.edu.cn and liu-ping@csco.org.cn. Telephone: +86 139 1389 4911, +86-25-68136428, +86-25-68136930; Fax: +86 025 68136099.

Table S1. Differently expressed miRNAs in the screening phase

**MiRNA**	**Fold change**			**Mean fold**
	Pool1	Pool2	Pool3	
miR-103	5.95	6.07	8.58	6.87
miR-106a	3.17	4.38	5.33	4.29
miR-106b	3.86	7.15	7.13	6.05
miR-107	4.86	6.64	6.93	6.14
miR-151-5p	4.31	7.30	12.03	7.88
miR-15b	2.77	3.62	5.55	3.98
miR-17	4.85	4.43	9.33	6.21
miR-182	4.02	3.24	15.15	7.47
miR-185	2.68	4.39	5.19	4.09
miR-19a	3.88	2.52	8.14	4.84
miR-20a	3.00	4.02	6.38	4.47
miR-21	4.81	5.84	12.16	7.61
miR-210	2.04	4.68	3.97	3.56
miR-223	3.13	4.51	10.63	6.09
miR-23a	2.26	3.36	5.92	3.85
miR-25	1.88	2.75	2.48	2.37
miR-320a	3.62	3.90	4.53	4.02
miR-335	1.92	3.55	6.18	3.88
miR-421	3.15	4.01	12.73	6.63
miR-532-5p	3.67	1.87	2.86	2.80
miR-584	3.07	3.86	7.25	4.73
miR-652	2.46	2.88	3.66	3.00
miR-92b	2.05	1.67	3.91	2.54
miR-16	2.45	2.64	3.60	2.90
miR-128	3.09	4.90	8.42	5.47
miR-181a	5.89	7.39	9.54	7.61
miR-199a-5p	3.54	5.68	9.48	6.24
miR-26a	5.98	12.20	11.90	10.03
miR-320b	2.42	3.67	4.07	3.39
miR-125a-5p	-2.62	-4.27	-1.94	-2.95
miR-133b	-1.99	-2.29	-2.09	-2.13
miR-133a	-2.63	-3.30	-2.87	-2.93
miR-365	-1.72	-5.52	-3.99	-3.74

Table S2. Expression levels of miRNAs identified from screening phase but not passed through the training stage (presented as mean ±SD; fmol/L).

MiRNA	Controls	Cases	FC	P value
miR-106a	604 ±190	1985 ±1767	3.29	0.10
miR-107	412 ±403	492 ±300	1.19	0.52
miR-128	985 ±1062	835 ±807	0.85	0.94
miR-133a	156 ±235	36.4 ±28.3	0.23	0.49
miR-133b	115 ±171	26.1 ±19.4	0.23	0.85
miR-151-5p	275 ±106	351 ±151	1.28	0.19
miR-15b	107 ±62.1	203 ±136	1.89	0.26
miR-16	14060 ±10829	21122 ±12051	1.50	0.20
miR-182	19.7 ±11.9	24.3 ±15.8	1.23	0.51
miR-199a-5p	44.7 ±24.2	105 ±120	2.35	0.52
miR-21	658 ±300	1358 ±1130	2.06	0.20
miR-223	35393 ±15857	56570 ±41424	1.60	0.34
miR-23a	947 ±413	1698 ±1455	1.79	0.77
miR-26a	1026 ±415	5084 ±5807	4.96	0.09
miR-320a	12069 ±3199	20990 ±18046	1.74	0.67
miR-320b	1302 ±319	2265 ±1763	1.74	0.90
miR-335	125 ±70.8	106 ±62.3	0.85	0.63
miR-365	352 ±562	33.9 ±33.7	0.10	0.89
miR-421	324 ±233	348 ±175	1.07	0.12
miR-532-5p	34.3 ±28.8	54.5 ±40.9	1.59	0.38
miR-584	158 ±118	345 ±377	2.18	0.76
miR-652	34 ±12.9	86.1 ±78.3	2.53	0.23

FC: fold change

Table S3. Expression levels of the other 6 miRNAs in the training and testing stages (presented as mean ±SD; fmol/L).

MiRNA	Training stage				Testing stage				Combined	
	Controls	Cases	FC	P value	Controls	Cases	FC	P value	FC	P value
miR-103-1	94.3 ±49.2	261 ±169	2.77	<0.001	88.6 ±51.3	97.2 ±79	1.10	0.23	1.55	0.58
miR-106b	543 ±290	1576 ±852	2.90	<0.001	1619 ±1556	1181 ±926	0.73	0.47	0.86	0.81
miR-17	972 ±797	1976 ±1248	2.03	0.003	972 ±670	1225 ±1018	1.26	0.79	1.48	0.32
miR-181a	22.5 ±13	72.7 ±56	3.23	<0.001	30.6 ±19.7	43.1 ±34.3	1.41	0.50	1.14	0.48
miR-19a	1097 ±1070	1957 ±1378	1.78	0.003	927 ±769	1047 ±805	1.13	0.13	1.35	0.15
miR-125a-5p	29.2 ±14.7	77.7 ±66.6	2.66	0.032	30.7 ±16.1	33.9 ±25.8	1.10	0.33	1.56	0.72

FC: fold change

Table S4. Expression levels of the five miRNAs in the external cohort (presented as mean ±SD; fmol/L).

MiRNA	Controls	Cases	FC	P value
miR-185	58.4 ±20.3	135 ±64.7	2.32	<0.001
miR-20a	2493 ±1003	5356 ±3739	2.15	0.046
miR-210	13.2 ±7.13	42.3 ±36.9	3.21	0.022
miR-25	925 ±363	2570 ±1881	2.78	0.004
miR-92b	9.2 ±6.4	44.4 ±28.4	4.82	<0.001

FC: fold change

Table S5. Analyses of the five miRNAs in the peripheral plasma compared to those in the arterial plasma (presented as mean ±SD; fmol/L).

MiRNA	Peripheral	Arterial	FC	P value
miR-185	128 ±79.8	56.3 ±29.9	0.44	<0.001
miR-20a	5646 ±4132	7720 ±4627	1.37	0.008
miR-210	36.8 ±27.6	86.4 ±94.5	2.35	0.013
miR-25	1781 ±1289	1754 ±919	0.98	0.14
miR-92b	31 ±20	103 ±93.7	3.34	<0.001

FC: fold change

Table S6. Expression levels of the five miRNAs in the peripheral exosomes of 10 GC patients and controls (presented as mean ±SD; △ Ct).

MiRNA	Controls	Cases	P value
miR-185	8.3 ±0.5	8.36 ±0.74	0.93
miR-20a	4.59 ±1.35	4.5 ±2.35	0.95
miR-210	7.2 ±0.75	7.87 ±0.998	0.37
miR-25	2.91 ±0.7	3.74 ±2	0.63
miR-92b	7.84 ±1.03	8.38 ±0.38	0.29

$\triangle Ct = Ct_{miRNA} - Ct_{cel\text{-}miR\text{-}39}$

Table S7. KEGG pathway analysis of the five identified miRNAs

**Analysis for *miR-185***	**P Value**	**Analysis for *miR-20a***	**P Value**	**Analysis for *miR-210***	**P Value**	**Analysis for *miR-25***	**P Value**	**Analysis for *miR-92b***	**P Value**
Long-term depression	0.003787292	Endocytosis	7.43059E-07	Glycosaminoglycan biosynthesis	0.002932615	FoxO signaling pathway	0.001326947	FoxO signaling pathway	0.000905768
Axon guidance	0.004581468	Bladder cancer	1.91037E-05	Hepatitis B	0.007958193	Proteoglycans in cancer	0.006364167	Proteoglycans in cancer	0.00423138
Arachidonic acid metabolism	0.005222847	MAPK signaling pathway	0.000051156	Prostate cancer	0.01244564	Circadian entrainment	0.007315897	Circadian entrainment	0.005575269
Glutamatergic synapse	0.008662666	Pathways in cancer	0.000146801	Cholinergic synapse	0.02346261	Focal adhesion	0.01283991	Regulation of actin cytoskeleton	0.01323466
Glutathione metabolism	0.008790736	TGF-beta signaling pathway	0.000571692	Neuroactive ligand-receptor interaction	0.02904973	Regulation of actin cytoskeleton	0.01862526	MicroRNAs in cancer	0.01627407
Ovarian steroidogenesis	0.008790736	Chronic myeloid leukemia	0.00075198	Cocaine addiction	0.03034573	PI3K-Akt signaling pathway	0.01909808	Focal adhesion	0.01936393
Adherens junction	0.009856015	Pancreatic cancer	0.000984466	Non-small cell lung cancer	0.03737776	Calcium signaling pathway	0.01991651	Nicotine addiction	0.02152255
Thyroid hormone signaling pathway	0.01003826	Axon guidance	0.001373651	Adrenergic signaling in cardiomyocytes	0.04737275	Nicotine addiction	0.02504522	RNA degradation	0.02738345
Ras signaling pathway	0.01178537	Circadian rhythm	0.002602499	Glioma	0.04896525	ECM-receptor interaction	0.02754333	Salivary secretion	0.02850573
T cell receptor signaling pathway	0.01556694	Prostate cancer	0.004736787			Small cell lung cancer	0.02754333	Dilated cardiomyopathy	0.02850573
VEGF signaling	0.01822438	mTOR signaling	0.004936159			Pantothenate and CoA	0.03231274	Pantothenate and	0.02918549

pathway		pathway				biosynthesis		CoA biosynthesis	
Fatty acid elongation	0.01917427	Melanoma	0.005791707			RNA degradation	0.03298968	Fc gamma R-mediated phagocytosis	0.03020303
Sulfur relay system	0.02482611	Hepatitis B	0.006385244			Adherens junction	0.03298968	Calcium signaling pathway	0.03128138
GnRH signaling pathway	0.02801417	Prolactin signaling pathway	0.006447754			Salivary secretion	0.03489878	Adrenergic signaling in cardiomyocytes	0.03842445
Notch signaling pathway	0.03194996	Glioma	0.008767568			Dilated cardiomyopathy	0.03489878	PI3K-Akt signaling pathway	0.03982208
Thyroid hormone synthesis	0.03444338	Small cell lung cancer	0.009396033			Fc gamma R-mediated phagocytosis	0.03692706	Pancreatic secretion	0.04405333
Endometrial cancer	0.0411774	Neurotrophin signaling pathway	0.01082131			MicroRNAs in cancer	0.04275079	Phosphatidylinositol signaling system	0.04464773
Vascular smooth muscle contraction	0.04684041	TNF signaling pathway	0.01167103			Amino sugar and nucleotide sugar metabolism	0.04613568	Glycosaminoglycan biosynthesis	0.04478341
		Transcriptional misregulation in cancer	0.01218648			Adrenergic signaling in cardiomyocytes	0.04876366		
		FoxO signaling pathway	0.01230724			Glycosaminoglycan biosynthesis	0.04940756		
		Regulation of actin cytoskeleton	0.01238419						
		Cell cycle	0.01439141						

	Renal cell carcinoma	0.0262943
	PI3K-Akt signaling pathway	0.02682305
	Non-small cell lung cancer	0.02744885
	Estrogen signaling pathway	0.02849083
	p53 signaling pathway	0.03124715
	HTLV-I infection	0.03405508
	Wnt signaling pathway	0.0363052
	Rap1 signaling pathway	0.03660535
	Protein processing in endoplasmic reticulum	0.04371815
	Osteoclast differentiation	0.0452136
	Dopaminergic synapse	0.0452136

Figure S1 Receiver-operating characteristic (ROC) curve analyses of each miRNA to discriminate GC patients from normal controls in the combined two cohorts

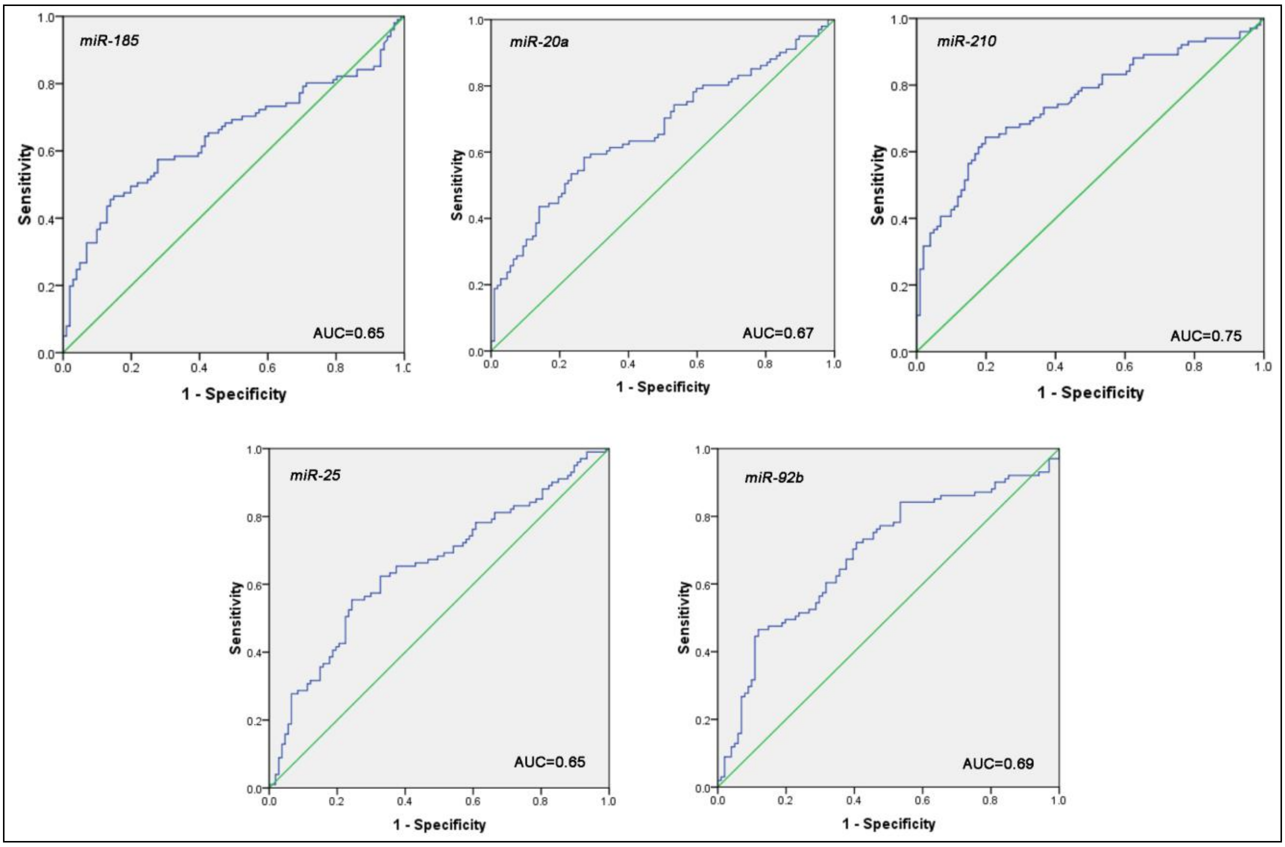

Supplement: Supplementary Information [file srep11251-s1.pdf]
